# A Six Years (2010–2016) Longitudinal Survey of the Four Serotypes of Dengue Viruses in Lao PDR

**DOI:** 10.3390/microorganisms11020243

**Published:** 2023-01-18

**Authors:** Charlotte Balière, Elodie Calvez, Jean-Michel Thiberge, Somphavanh Somlor, Mathias Vandenbogaert, Marc Grandadam, Valérie Caro

**Affiliations:** 1 Environment and Infectious Risks Unit, Institut Pasteur, 75015 Paris, France; 2 Arbovirus and Emerging Viral Diseases Laboratory, Institut Pasteur du Laos, Vientiane 01030, Laos

**Keywords:** dengue, DENV-1, DENV-2, DENV-3, DENV-4, envelope gene, phylogeny, Lao PDR

## Abstract

Dengue fever is the most prevalent arthropod-borne viral infection of humans in tropical and subtropical countries. Since 1979, dengue has been reported to be endemic in the Lao People’s Democratic Republic (PDR), as in many countries in Southeast Asia, with a complex circulation of the four dengue viruses’ serotypes (DENV-1 to DENV-4). By sequencing the complete envelope protein, we explored a panel of samples from five Lao Provinces (Vientiane capital, Luangprabang, Bolikhamxay, Saravane, Attapeu) to enrich knowledge about the co-circulation of DENVs in Lao PDR between 2010 and 2016. Phylogenetic analyses highlighted the specific circulation of DENV-1 genotype I, DENV-2 genotype Asian I, DENV-4 genotype I and the co-circulation of DENV-3 genotype II and III. The continuous co-circulation of the four serotypes was underlined, with genotype or cluster shifts among DENV-3 and DENV-1. These data suggested the emergence or re-emergence of DENV strains associated with epidemic events, potentially linked to the exchanges within the territory and with neighboring countries. Indeed, the increasing local or regional connections favored the dissemination of new isolates or new clusters around the country. Since 2012, the surveillance and alert system created in Vientiane capital by the Institut Pasteur du Laos appears to be a strategic tool for monitoring the circulation of the four serotypes, especially in this endemic country, and allows for improving dengue epidemiological knowledge to anticipate epidemic events better.

## 1. Introduction

Dengue fever is the most prevalent human arthropod-borne viral infection in tropical and subtropical countries, further expanding due to climate change and urbanization [1]. Already endemic in more than 100 countries [2,3], this arboviral disease can cause a panel of clinical forms ranging from self-resolving flu-like illness syndromes with or without warning signs to severe dengue with plasma leakage, bleeding or organ impairment evolving to death [4]. Given the number of exposed people around the world (around 3.9 billion) and its potentially severe clinical forms, this disease poses a significant public health and economic burden in endemic areas and in Northern hemisphere countries where dengue viruses (DENV) and its *Aedes* spp. vectors extended [2,5].

The etiological agent of this disease, *Dengue virus* (DENV), belongs to the *Flavivirus* genus, *Flaviviridae* family. DENV genome consists of a single-stranded, positive-sense RNA and contains a long open reading frame of 10,700 nucleotides encoding for three structural proteins, capsid (C), pre-membrane (prM) and envelope (E), and seven non-structural proteins (NS1, NS2A, NS2B, NS3, NS4A, NS4B and NS5). DENV displays four antigenically distinct serotypes (DENV-1, DENV-2, DENV-3 and DENV-4), exhibiting more than 30% divergence along the overall amino-acid sequence [6,7]. The antigenic distance is sufficient to explain the lack of long-term cross-protective immunity against the three others [8]. Interestingly, genetic distances recorded within the envelope protein gene are in good correlation when compared with full genome data sets. Genotypes are often related to specific geographical regions, and clusters within genotypes provide a deeper degree of molecular epidemiology as they determine topotypes [9,10]. A higher risk of severe dengue has been linked to the co-circulation of multiple serotypes and/or genotypes due to antibody-dependent enhancement of infection and some specific isolates [11,12]. These observations demonstrate the importance of developing and maintaining robust surveillance networks to follow the circulation of serotypes and the potential introduction of new genotypes in endemic regions.

Dengue outbreaks have been reported in all countries in Southeast Asia, including China, Myanmar, Thailand, Cambodia and Vietnam [13] and in the low–middle-income country of the Lao People’s Democratic Republic (PDR) [11]. Since the first dengue hemorrhagic fever (DHF) cases in 1979 were reported in the country [14], dengue disease is considered to be endemic in Lao PDR with the circulation of the four DENV serotypes [15,16,17,18,19,20]. In 2008 and in 2013, two major dengue outbreaks were described in the country, respectively, due to DENV-1 (in the northwestern part of the country) and DENV-3 (at the country level) [15,16]. In 2014, an increased number of DENV-4 cases was recorded, followed by a major outbreak in Lao PDR [20]. A dengue surveillance and alert system based on a weekly recording of the dengue-like syndrome has been in place in Lao PDR since 2006 [21]. In 2012, the setting up of a complement network of dengue diagnosis capacities at Vientiane capital by the Institute Pasteur of Lao PDR (IPL) allowed to improve dengue surveillance and epidemiological situation knowledge in this country of 7 million people (www.lsb.gov.la; accessed on 1 September 2022), among which nearly 41% are less than 20 years old in 2020 [16,20,22].

Our aim was here to provide phylogenetic analyses of the four DENV serotypes in Lao PDR between 2010 and 2016 to decipher the complexity of dengue epidemiology in the country in the context of the active DENV circulation at the regional scale.

## 2. Materials and Methods

### 2.1. Ethics Statement

The study protocol was submitted and approved by the National Ethic Committee for Health Research of the Ministry of Health of Lao PDR (N°49/NECHR and N°2018.116). All public hospitals’ management committees approved the study and obtained the agreement of the Ministry of health to participate in the protocol. All adult volunteers provided written informed consent. A parent or legal guardian of any child included in the study signed a consent form on their behalf.

### 2.2. Human Samples Collection

**Figure 1:**
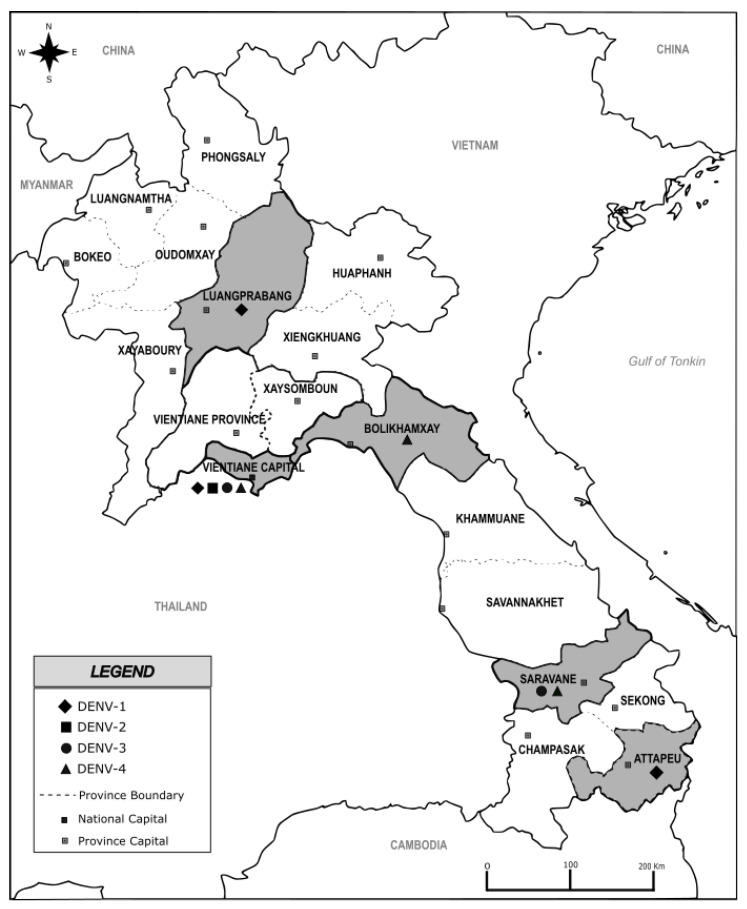
Map of Lao PDR showing sampling provinces highlighted in grey, investigated from 2010 to 2016. The black marks diamond, square, round and triangle highlight the serotype detected in each sampling province. Map was created using Inskape^®^ software (version number 0.92.5), free of copyright.

At the beginning of the survey in June 2010, samples were collected by the Centre Médical de l’Ambassade de France with the dengue surveillance based on a weekly recording of the dengue-like syndrome in place in Lao PDR since 2006. Between February 2012 and July 2016, samples were collected continuously through the setting up of a complement network of dengue diagnosis capacities at Vientiane capital coordinated by the Institut Pasteur du Laos. Suspected dengue fever cases were selected according to the WHO’s case definition (fever onset ≥ 38 °C for less than 7 days with at least one of the following accompanying symptoms: headache, myalgia, arthralgia, retro-orbital pain, digestive troubles or hemorrhaging) [4]. In this survey, the sequenced samples were selected from a biobank of samples collected in five Provinces covered by the surveillance network, i.e., Luangprabang, Bolikhamxay, Vientiane capital, Attapeu and Saravane (Figure 1). The blood (5 mL) obtained by venipuncture on EDTA tubes was stored at 4 °C during transportation to the Institut Pasteur du Laos until diagnosis and molecular investigation.

### 2.3. Dengue Viruses Screening and Viral RNA Extraction

Samples were screened for the presence of the DENV genome using a pan-dengue real-time RT-PCR [23], and serotypes were determined by a specific real-time RT-PCR [24]. 

In case of a very low viral load attested by a Ct value above 30, plasma was inoculated on C6/36 cell monolayers [25]. Total viral RNA was extracted either directly from Human plasma or from the supernatant of C6/36 infected cultures. Extractions were carried out using the NucleoSpin II RNA kit (Macherey Nagel) according to the manufacturer’s instructions.

### 2.4. Envelope Gene Sequencing

Envelope gene sequencing was performed on a panel of 78 samples according to their geographical origin, the year of collection, the Ct value for the pan-dengue real-time RT-PCR and the remaining sample volume. Specific amplification was performed with sets of primers, specifically designed according to the serotype, in order to produce three or four overlapping amplicons (Table 1). Amplicons were generated using the SuperScript One-Step RT-PCR with PlatiniumTaq kit (Invitrogen), as previously described [15]. Following a 1% agarose gel electrophoresis, PCR products were purified using the NucleoFast kit (Macherey Nagel) as specified by the manufacturer. Sequencing was carried out using the BigDye™ Terminator v1.1 Cycle Sequencing Kit (Applied Biosystems). The sequencing reaction was performed in a volume of 10 µL containing 2 µL of purified PCR product template, 4 µL of ddH2O, 1 µL of sequencing buffer (5×), 1 µL of sense primer or anti-sense primer (4 µM) and 2 µL of Big Dye 1.1. The sequencing program was performed as follows: 96 °C 1 min followed by 30 cycles of 96 °C 10 s, 50 °C 5 s, 60 °C 1 min 15 s. The sequencing reactions were purified using the BigDye XTerminator™ Purification Kit according to the manufacturer’s instructions. Sequence chromatograms for both strands were obtained using an automated analyzer, ABI3730xl (Applied Biosystems, Waltham, MA, USA).

### 2.5. Phylogenetic Analysis

Nucleotide sequences of the complete E gene obtained in this study were submitted to EMBL-EBI, and their accession numbers (MN628181 to MN628258) are detailed in Table 2. The complete E gene of Lao DENV isolates was edited using the software BioNumerics V7.6 (Applied-Maths, Saint-Martens-Latem, Belgium).

A first step of sequence analysis and comparison was performed with all available DENV envelope sequences extracted from GenBank. For clarity, only a subset of envelope sequences within each serotype was used for the final phylogenetic analysis after removing unsuitable or redundant sequences but keeping representatives of each genotype and study area. Additionally, the previously published Lao sequence data set was integrated for phylogenetic analyses for consistency [16,19,20,22].

For each DENV serotype, multiple sequence alignment of the E gene was obtained using MAFFT version 7.023b [26] after removing the duplicated sequences. The unrooted phylogenetic trees were constructed using RAxML [27] with a general time-reversible plus gamma distribution substitution model and a rapid bootstrap (i.e., model GTR  +  I  +  G, bootstrap  =  1000) and visualized in FigTree version 1.4.3). For the phylogenetic analyses, the data set, respectively, included a panel of 84, 58, 53 and 57 Envelop gene sequences of, respectively, DENV-1, DENV-2, DENV-3 and DENV-4 retrieved from GenBank Database (https://www.ncbi.nlm.nih.gov/genbank/; accessed on 12 August 2022).

## 3. Results

### 3.1. Serotype Circulation of DENV during 2010–2016 in Lao PDR

Among the 4546 samples tested between 2010 and 2016, 2143 were found positive for DENV by RT-PCR, and 1103 samples could be serotyped. The mean age of the patients infected by DENV was 20.67 years old (4 months to 86 years old). After 2015, the data collected indicated a sex ratio of 52.4% of males and 47.6% of females (N = 646). During the same period, for the patients found positive by RT-PCR, the clinical diagnosis clustered the patients as 634 dengue fever, 9 dengue hemorrhagic fever and 3 dengue shock syndromes. Among those 646 samples, 10 fatal cases were recorded (DENV-1: 1; DENV-2: 1; DENV-3: 5; DENV-4: 1 and unknown DENV: 2). Since three different serotypes have been associated with fatal cases, it is therefore difficult and risky to draw a direct link between a severe case and a specific serotype or genotype. The severity is more likely a consequence of the deferred patients’ management.

**Figure 2:**
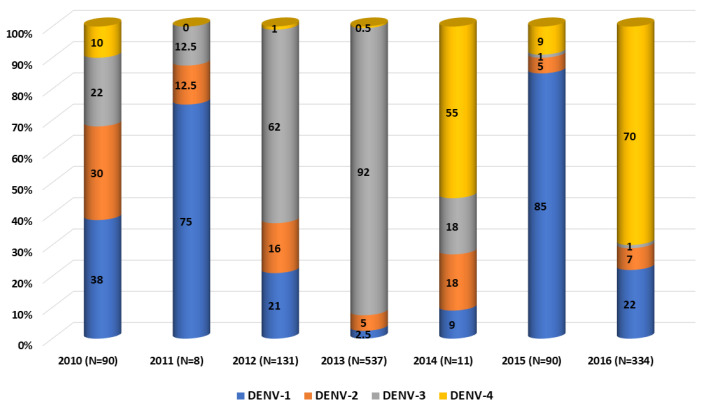
Dengue virus serotypes distribution per year between 2010 and 2016. Compilation of data from 2010 to 2011 [21] and from 2012 to 2016 [20].

The four DENV serotypes were detected from 2010 to 2016; nevertheless, a predominant serotype has alternated during the study period. Together with previously published data on DENV serotype circulation in Lao PDR [20,21], DENV-1 was dominant in 2010–2011 and in 2015, DENV-3 in 2012–2013 and DENV-4 in 2014 and 2016 (Figure 2). Samples analyzed in this study were collected all year long, with 75% of samples obtained between August and November, consistent with previous observations during the rainy season and the increase in vector density [16,18].

### 3.2. DENV Sequences Analysis

A subset of 78 complete coding sequence (CDS) envelope genes was sequenced (1485 nt for DENV-1, DENV-2 and DENV-4; 1479 nt for DENV-3) and was investigated to complete the overview of the circulation of the four DENV serotypes circulating in Lao PDR from 2010 to 2016: 51 DENV-1 (from Luangprabang, Attapeu and Vientiane capital), 21 DENV-2 (from Vientiane capital), 2 DENV-3 (from Saravane and Vientiane capital) and 4 DENV-4 (from Bolikhamxay, Saravane and Vientiane capital) (Table 2).

In order to compare in a consistent way our sequence data with previously published results and to better understand the molecular epidemiology within Lao PDR, we have considered the definition of the local clusters already described and have thus completed the description of their dynamics with our 2010–2016 Lao PDR series [10,15,16,18,20].

**Figure 3:**
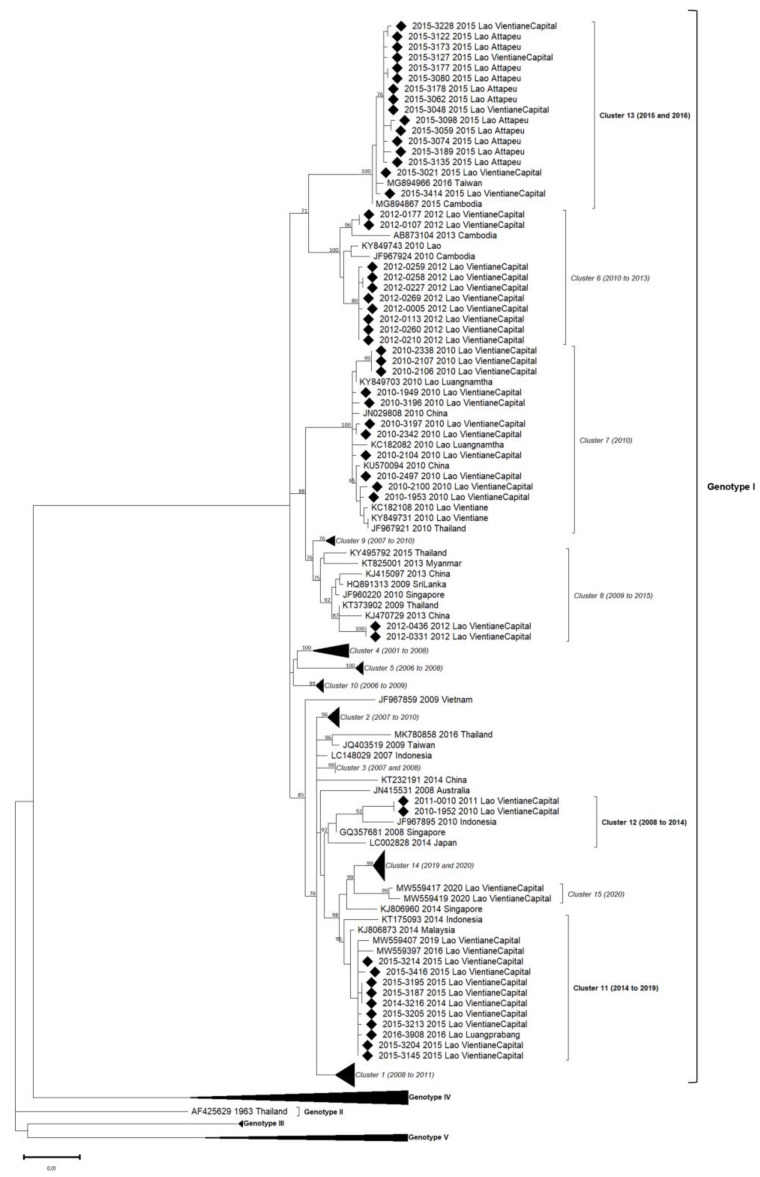
Phylogenetic analysis of DENV-1: 84 complete CDS protein E references of DENV-1 were selected from GenBank and aligned with the 51 DENV-1 sequences from this study (indicated with black diamond). Multiple sequence alignments were obtained using MAFFT version 7.023b. The unrooted phylogenetic trees were constructed using RAxML with general time-reversible plus gamma distribution substitution model and a rapid bootstrap (i.e., model GTR  +  I  +  G, bootstrap  =  1000). The major genotypes and clusters in which sequences from this study are grouped are indicated. Statistical support values of grouping (maximum-likelihood bootstrap replicates >60%, as calculated by RAxML) are indicated at nodes of the tree. Scale bar indicates nucleotide substitution per site. Clusters in italics (C1 to C10) were predefined by Castonguay-Vanier et al., 2018, and Calvez et al., 2021, and reported in this phylogenetic tree. Clusters in bold were defined in this study (C11 to C13) [18,22].

**Figure 4:**
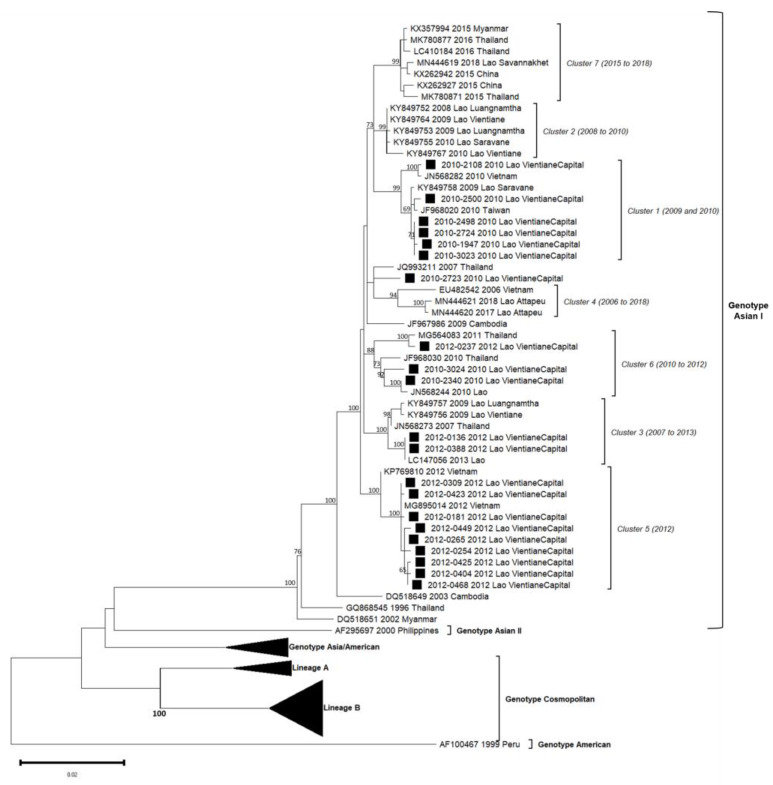
Phylogenetic analysis of DENV-2: 58 complete CDS protein E references of DENV-2 were selected from GenBank and aligned with the 21 DENV-2 sequences from this study (indicated with black square). Multiple sequence alignments were obtained using MAFFT version 7.023b. The unrooted phylogenetic trees were constructed using RAxML with general time-reversible plus gamma distribution substitution model and a rapid bootstrap (i.e., model GTR  +  I  +  G, bootstrap  =  1000). The major genotypes, lineages, and clusters in which sequences from this study are grouped are indicated. Statistical support values of grouping (maximum-likelihood bootstrap replicates > 60%, as calculated by RAxML) are indicated at nodes of the tree. Scale bar indicates nucleotide substitution per site. Clusters in italics (C1 to C6) were predefined by Castonguay-Vanier et al., 2018, and Calvez et al., 2020, and reported in this phylogenetic tree [18,19].

**Figure 5:**
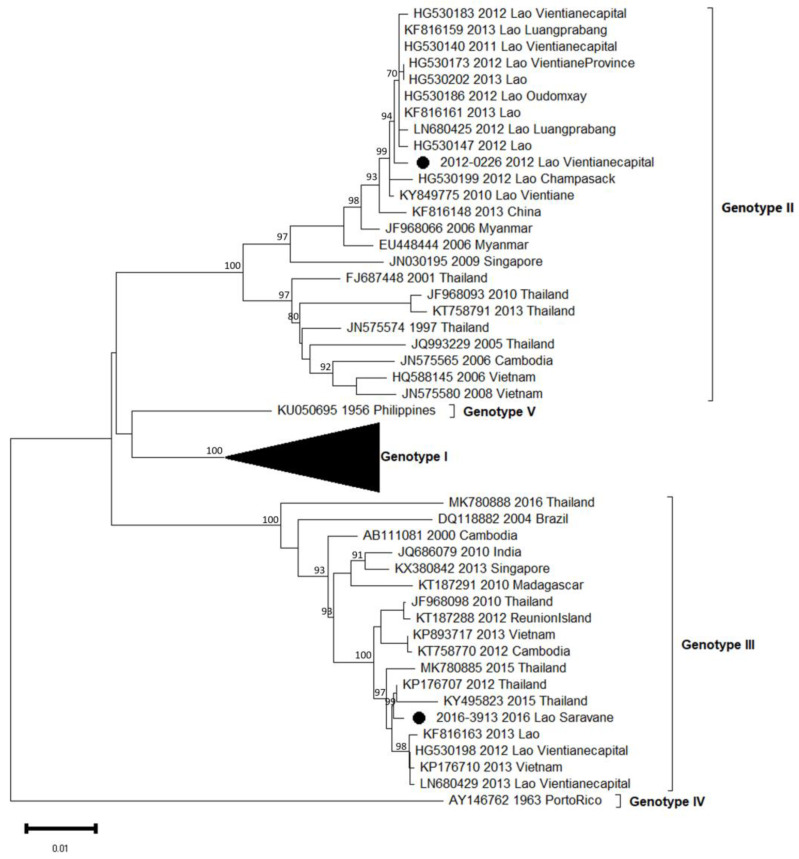
Phylogenetic analysis of DENV-3: 53 complete CDS protein E references of DENV-3 were selected from GenBank and aligned with the 2 DENV-3 sequences from this study (indicated with black circle). Multiple sequence alignments were obtained using MAFFT version 7.023b. The unrooted phylogenetic trees were constructed using RAxML with general time-reversible plus gamma distribution substitution model and a rapid bootstrap (i.e., model GTR  +  I  +  G, bootstrap  =  1000). The major genotypes and lineages in which sequences from this study are grouped are indicated. Statistical support values of grouping (maximum-likelihood bootstrap replicates >60%, as calculated by RAxML) are indicated at nodes of the tree. Scale bar indicates nucleotide substitution per site.

**Figure 6:**
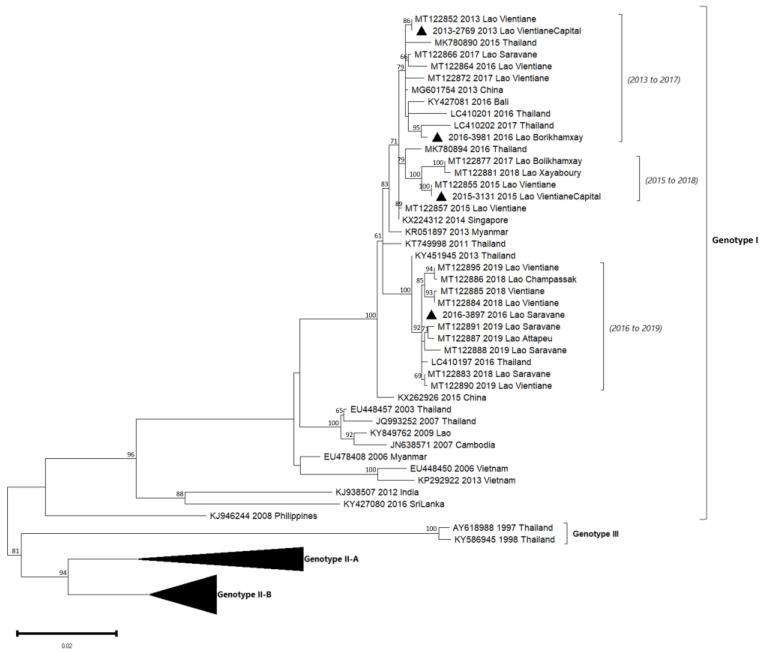
Phylogenetic analysis of DENV-4: 57 complete CDS protein E references of DENV-4 were selected from GenBank and aligned with the 4 DENV-4 sequences from this study (indicated with black triangle). Multiple sequence alignments were obtained using MAFFT version 7.023b. The unrooted phylogenetic trees were constructed using RAxML with general time-reversible plus gamma distribution substitution model and a rapid bootstrap (i.e., model GTR  +  I  +  G, bootstrap  =  1000). The major genotypes and lineages in which sequences from this study are grouped are indicated. Statistical support values of grouping (maximum-likelihood bootstrap replicates > 60%, as calculated by RAxML) are indicated at nodes of the tree. Scale bar indicates nucleotide substitution per site. Lineage (a), predefined by Hamel et al., 2019, was reported in the phylogenetic tree [10].

Phylogenetic trees showed in this analyzed subset that the sequences obtained for DENV-1, DENV-2 and DENV-4 belonged to a unique genotype, i.e., respectively, genotype I, Asian I genotype and genotype I (Table 2, Figure 3, Figure 4 and Figure 6). Over the period studied, a co-circulation of two genotypes was only found for DENV-3 with the genotypes II and III (Table 2, Figure 5).

### 3.3. DENV-1 Phylogeny

The 51 DENV-1 envelope gene sequences were obtained from a panel of samples collected in 2010, 2011, 2012, 2014, 2015 and 2016 (Table 2). During this six years period, DENV-1 circulation was uninterrupted.

Within DENV-1 genotype I, the 51 sequences were distributed in six different clusters: three of them have already been described (clusters 6, 7 and 8), and three others were identified in this study (clusters 11, 12 and 13) (Figure 3). All genotype I clusters presented below 3% nucleotide divergence between them. 

Among isolates collected in 2010, eleven were grouped in cluster C7 and were closely related to DENV circulating at the same period in Luangnamtha Province (North of Lao PDR), in Vientiane capital [15,18] and in neighboring countries such as China and Thailand. The isolates belonging to the C7 shared above 99.5% of nucleotide identity with each other (99.6% of amino-acid identity), and the cluster was strongly supported by bootstrap value. Ten DENV-1 sequenced from 2012 in Vientiane capital grouped in C6. They displayed a high nucleotide identity percentage with older isolates from Lao PDR (2010) and Cambodia (2010, 2013) (99.4%) and 99.5% of amino-acid identity. Two DENV-1, isolated in 2012 in the Vientiane capital, grouped in C8 with isolates detected in Thailand (2009) and in China (2013). Even if C8 gathered strains identified over a broad time frame, the percentage of nucleotide identity significantly remained very high (98.8%) with 99.7% of amino-acid identity.

Nine sequences obtained in 2014 and 2015 in the Vientiane capital and one in 2016 in Luangprabang shared 99.8% nucleotide identity (100% amino-acid identity) with isolates identified in 2014 in Malaysia, Indonesia and Singapore. Thus, this group of isolates defined the new cluster, namely C11, supported by bootstrap value.

Another pair of DENV-1 isolates collected in the Vientiane capital in 2010 and 2011 were closely related to an Indonesian strain isolated in 2010, sharing 100% amino-acid identity between them and 98.9% of nucleotide identity in the cluster, well supported by bootstrap value, defining a new cluster namely C12.

Sixteen DENV-1, isolated in Attapeu and Vientiane capital in 2015, shared 99.7% of nucleotide identity with each other and grouped with 2015 Cambodian and 2016 Taiwanese sequences. Altogether, this panel of isolates determined the last new cluster, namely C13 (with 99.8% amino-acid identity), supported by a strong bootstrap value.

DENV-1 sequences in this study were linked to six distinguished clusters closely related to a timeline belonging to genotype I. For each year, 2010, 2012 and 2015, two clusters were co-circulated: respectively, C7/C12, C6/C8 and C11/C13. All the circulating clusters were represented in Vientiane capital. 

### 3.4. DENV-2 Phylogeny

A total of 21 DENV-2 isolates (2010: n = 9, 2012: n = 12) were successfully sequenced from positive patients from the Vientiane capital. Up to four distinct clusters of DENV-2 previously defined among the Asian I genotype (C1, C3, C5 and C6) were identified over this two years period in this sole province (Figure 4).

C1 only gathered isolates from 2010 (n = 6) with high bootstrap values. Lao isolates were closely related to isolates from Taiwan (2010) and Vietnam (2010) and a Lao isolate from Saravane (2009), a province in Southern Lao [18]. The percentage of nucleotide identity observed in C1 isolates reached 99.3% (99.9% of amino-acid identity).

Two DENV-2 from 2012 were congregated in C3 and displayed sequences that fully matched with a 2013 Lao sequence (100% nucleotide identity between these three isolates and strongly supported by high bootstrap value). This cluster also encompassed Lao isolates from 2009 from the Vientiane capital and Thai isolate from 2007.

A set of nine DENV-2, all detected between August and December 2012, were classified in the C5. Sequences from Lao PDR included in this cluster displayed an overall nucleotide identity above 99.5% (99.9% of amino-acid identity), shared with the isolate of a Taiwanese traveler who arrived from Vietnam in 2012 (MG895014) and one isolate from Vietnamese autochthonous case in 2012 (KP769810).

C6 gathered two DENV-2 isolates from 2010 and one from 2012, all from the Vientiane capital (nucleotide identity of 98.6%). Interestingly, Lao isolates from 2010 were linked to Lao and Thai isolates from 2010, whereas the 2012 isolates matched with a Thai strain from 2011.

The DENV-2 isolate number 2010–2723 shared 99.1% of nucleotide identity with the 2007 Thai strain but has remained unrelated to a defined cluster.

Overall, for the DENV-2 series, only the Asian I genotype is represented between 2010 and 2012. However, isolates were distributed into four clusters, grouping isolates circulating in Lao PDR and in neighboring countries, especially Taiwan, Vietnam and Thailand, between 2007 and 2013. In 2010 two clusters (C1/C6) and three in 2012 (C3/C5/C6) co-circulated but only C6 persisted over time.

### 3.5. DENV-3 Phylogeny

During the 2012/2013 epidemic of dengue in the Vientiane capital, we have shown that a major serotype switch occurred from DENV-1 to DENV-3, with the latter serotype dominant at 92% in 2013 [16,20]. The previous study of Lao et al. also evidenced the co-circulation of two DENV-3 genotypes, i.e., genotypes II and III, probably introduced in an independent and singular manner. Here, we completed our previous data set with two supplementary autochthonous isolates, one from Vientiane capital in 2012 and one more recent from Saravane in 2016, representing the two main circulating genotypes (Table 1, Figure 5). As expected, the 2012 isolate belonged to genotype II and grouped with the previous ones described between 2010 and 2013 in five different Lao provinces, i.e., Champasak, Luangprabang, Oudomxay, Vientiane capital and Vientiane province [15]. The second isolate from 2016 belonged to genotype III, but intriguingly, it was found to be more closely related to regional isolates such as those from Cambodia (2012), Vietnam (2013) and Thailand (2010 to 2016), sharing 98.9% identity. Considering the work published by Lao et al. in 2014 [16] focused on the circulation of DENV-3 in the Lao PDR, genotype III emerged in late 2012 and then remained dominant in 2013.

### 3.6. DENV-4 Phylogeny

Between 2010 and 2014, DENV-4 was the least detected in comparison with the other serotypes in Lao PDR. Indeed, this serotype represented 10% in 2010 and <1% in 2013 of the dengue isolates (Figure 2). The four isolates included in our series were from 2013 (n = 1), 2015 (n = 1) and 2016 (n = 2) from Vientiane capital, Saravane and Bolikhamxay (Table 2, Figure 6). Three of four of the DENV-4 sequenced displayed an overall nucleotide identity above 98.5% (99.4% of amino-acid identity), with strains circulating in bordered countries between 2013 and 2018 and grouped with fatal dengue cases in Lao and Thailand occurred between 2015 and 2017 (Figure 6). All the determined DENV-4 sequences in this study were not fully related to the first reported presence of DENV-4 in Lao in 2009, identified in Saravane province (3.6% of nucleotide divergence) [18].

## 4. Discussion

The Indochinese peninsula is considered an area of hyper-endemicity for dengue fever [13], within which Lao PDR occupies a central geographic position. This topographic specificity exposes the country to potential emergence or re-emergence events of DENV serotypes/genotypes, promptly leading to epidemic situations [16,20,28]. Some studies have described the epidemiological situation in this country, either through a longitudinal approach, such as the 2006–2010 study [18], or by describing an epidemic linked to a single serotype, such as the study of molecular epidemiology of the dynamics of DENV-3 between 2012 and 2013 [16], the rapid genotyping protocol investigated on DENV-2 in 2018 [19], the trends in the circulation of DENV-4 between 2015 and 2019 [20], and the prospective study on DENV-1 circulation in Lao PDR [22]. Through the weekly recording of dengue-like syndrome since 2006, combined with the setting up of a hospital-based network in the Vientiane capital with laboratory diagnostic capacities provided by the Institut Pasteur du Laos since 2012, we conducted a six-year longitudinal study (2010–2016). The aim was to provide an additional global spatio-temporal point of view of DENV molecular epidemiology in the Lao PDR. The capital Vientiane represents 10% of the total population of the country and is a good proxy to estimate the global epidemiological situation, due to, among other things, the main international hub for the country and thecentralization of the university system. Advanced surveillance capabilities for dengue are up to now concentrated in Vientiane Capital city. Thus, the efficiency of the surveillance network directly depends on logistic issues of the shipment of biological samples from provinces to the capital city. Therefore, even if the number of DENV sequences was limited, the heterogeneous geographical and temporal distribution of the selected samples for sequencing during this timeline allowed us to have an overview of the four serotypes’ circulation in the Lao PDR. 

A majority of DENV viruses were detected during the third trimester corresponding to the peak of the rainy season in Lao PDR [16,18,20]. Although we only analyzed samples from 5 of the 18 Lao provinces, all four DENV serotypes were detected both in the northern and southern parts of the country, as it has been previously demonstrated since 1987 [14,18,29]. Five peaks of detection of the DENV could be noticed: 2011 with a predominance of DENV-1 serotypes, 2012–2013 with DENV-3, 2014 with DENV-4, 2015 with DENV-1 followed by DENV-4 in 2016.

Over our study, DENV-1 was the most frequently detected serotype along the 6-year survey, with notably the largest dynamic range of evolution with many identified clusters. The single genotype detected (genotype I) could be split into thirteen clusters, with the description of three new ones. During the same year, several clusters were circulating, for example, in 2015 with the clusters C11 and C13, in 2012 with C6 and C8, and in 2010 with C7 and C12. In addition, some clusters seemed to be associated with geographical areas, such as the C6 and C11 clusters in the capital Vientiane and the C13 cluster in Attapeu, according to the sampling information available until now. Both these observations highly suggested a spatio-temporal introduction of DENV-1 in Lao PDR between 2010 and 2016, as also observed by Calvez et al. [22]. Over this period, DENV-1 was circulating in the north and the south of the country, accounting for almost 85% of the serotyped samples [15,18]. In 2008, DENV-1 could be linked to an epidemic event in a remote rural village in Xayaboury province [15]. At the end of 2010, DENV-1 was dominant in Vientiane capital but also in the Northeast of Thailand, a bordered country [30], suggesting probable epidemiological exchanges between the two countries.

In Lao PDR, the serotype DENV-2 was still present between 2010 and 2016, even at low levels for some years. In the present study, only isolates from the Asian I genotype were detected and sequenced, segregating them into already-known clusters. Although exclusively detected in the Vientiane capital in this study, the Asian I genotype has been further described since 2016 in other Lao provinces (Attapeu and Saravane), highlighting the ongoing circulation over the country [19]. Globally, Asian I, Asian/American and Cosmopolitan genotypes have been documented as co-circulating in Thailand, Cambodia or Vietnam [10,31,32]. Previously published data have shown that DENV-2 continued to circulate well beyond 2012. Since 2017, Asian I and Cosmopolitan genotypes (lineages A and B) were both detected in different Lao PDR provinces, such as Vientiane capital and Vientiane province, Luangprabang and Attapeu until 2019 [19]. According to the serotype distribution (Figure 2), DENV-2 was the least frequently identified serotype in Lao PDR between 2010 and 2016 but seriously increased after 2017 [19].

As previously published, in 2012/2013, Lao PDR faced a major DENV-3 epidemic, during which we could evidence the co-circulation of two genotypes, II and III [16]. After the 2012–2013 outbreak, DENV-3 was less detected and totally absent after 2016, according to the published data from the surveillance and alert system created in Vientiane capital by the Institut Pasteur du Laos [22].

Between 2010 and 2013, DENV-4 samples represented less than 10% of the total number of serotype determinations in the country. DENV-4, collected in 2013 and 2015, were identified in the Vientiane capital, followed by isolates from 2016 in more remote provinces. These results are consistent with previous analysis: since 2014, cases started to be recorded in Vientiane city intra muros, followed three years after by an outbreak that peaked between June and August 2017 at the country level [20].

Overall, in our phylogenetic analyses, even if some branches were weaker supported, all the clusters identified in each serotype were still well supported by bootstrap values. In order to obtain a better phylogenetic signal, it would be interesting to analyze a larger number of samples over a longer period.

Our study, conducted from 2010 to 2016, confirmed multiple potential introductions, emergence or re-surgency with the spread of the four serotypes between the north and the south of Lao PDR. All the connections, with an active circulation of people throughout the country or the Indochinese peninsula, probably favored the dissemination or the introduction of new isolates or clusters among Lao PDR. Lao population, with limited prior DENV exposure, could thus be exposed to severe or even fatal diseases [15,31,33,34,35].

Indeed, like its neighboring countries, Lao PDR presents a complex dynamic of DENV circulation, both in terms of serotype and geographical origin. Emergence, importation, introduction, resurgence, co-circulation, evolution, spread and switch are all words used in the description of dengue events in Southeast Asian countries [1,9,28,31,32,33,34,35]. The geographical context of Lao PDR, associated with the development of tourism and the commercial exchanges between provinces and the capital but also between the countries at the regional scale or beyond, could be the source of the spread of the four dengue serotypes.

The circulation of DENV poses a challenge to Lao PDR public health authorities, especially since the disease has been recognized as a major public health issue in the country [14,21]. Vientiane capital is a strategic monitoring site with the most extensive collection site for human specimens, allowing numerous epidemiological studies [19,20,22] and where all four serotypes were continuously detected. Finally, in the global era of whole genome sequencing, generating only the envelop gene sequence is proved to be sufficiently informative and robust to monitor the genomic surveillance of dengue. This is even more important as in low- and middle-income countries such as Lao PDR, implementing next-generation sequencing, requiring significant laboratory infrastructure and computational capacity, remains a real challenge.

## Figures and Tables

**Table 1:** Primers used for the sequencing of envelope gene E.

Dengue Type		Primer Name	Sequence (5′-3′)	Size of Amplicon (bp)	Reference
DENGUE 1	DENV1-FG1	Den1-771F	CCTCTGAAGGCGCTTGGAA	780	This study
		Den1-1551R	CCATTGTTTGTGGACGAGCC		This study
	DENV1-FG2	Den1-1112F	TGCATTGAAGCCAAAATATCAAA	1442	This study
		Den1-2554R	CTCCCATGCCTTCCCAATG		This study
	DENV1-FG3	Den1-2189F	GCATGGGACTTCGGCTCTATAGG	1293	This study
		Den1-3482R	CTGACCCTGCAGAGACCATTGA		This study
DENGUE 2	DENV2-FG1	Den2-279F	AACAGCAGGGATATC	1389	This study
		Den2-1668R	ATGGCGATTTTTGAAACTCA		This study
	DENV2-FG2	Den2-1137F	CTGTATAGAAGCAAAGCTGACC	610	This study
		Den2-1747R	GTAAGTTTCCTGATGACATC		This study
	DENV2-FG3	Den2-1860F	TGTGAAGGAAATAGCAGA	599	This study
		Den2-2459R	AGTTCTTTGTTTTTCCAGCT		This study
	DENV2-FG4	Den2-2297F	TTATGAAAATCCTCATAGGA	1209	This study
		Den2-3506R	AGTGAAAAGTTGTCAATCTG		This study
DENGUE 3	DENV3-FG1	Den3-1F	AGTTGTTAGTCTACGTG	1012	[16]
		Den3-1013R	GGTAGTCACACACCCCCCGTG		[16]
	DENV3-FG2	Den3-815F	GCCCTTAGGCACCCAGGGTT	937	[16]
		Den3-1752R	CCCGCGAAAATGCTTGTGC		[16]
	DENV3-FG3	Den3-1398F	CGCAAGGAGTCACGGCTGAG	1141	[16]
		Den3-2539R	GCCTGCAATGGCTGTTGCC		[16]
DENGUE 4	DENV4-FG1	Den4-848F	GGCAGGATTTATGGCTTACATG	640	This study
		Den4-1488R	CGAGTGTTAGTTCTCCATAG		This study
	DENV4-FG2	Den4-1398F	GACACATCCAATCATGGAG	686	This study
		Den4-2084R	AACACCTATCACTATGTAGC		This study
	DENV4-FG3	Den4-1954F	TCCCCATAGAGATAAGAGATG	661	This study
		Den4-2615R	GGTTGATCTAATTCCACAG		This study

**Table 2:** List of studied Lao Dengue isolates selected during the longitudinal survey from 2010 to 2016.

Key ^1^	Location		Month-Year of Collection	Serotype	Genotype	GenBank Accession Number
	Country	Province				
2010-1949	Lao PDR	Vientiane capital	Sept-2010	DENV-1	I	MN628181
2010-1952	Lao PDR	Vientiane capital	XX ^2^-2010	DENV-1	I	MN628182
2010-1953	Lao PDR	Vientiane capital	Sept-2010	DENV-1	I	MN628183
2010-2100	Lao PDR	Vientiane capital	Sept-2010	DENV-1	I	MN628192
2010-2104	Lao PDR	Vientiane capital	Sept-2010	DENV-1	I	MN628193
2010-2106	Lao PDR	Vientiane capital	Sept-2010	DENV-1	I	MN628194
2010-2107	Lao PDR	Vientiane capital	Sept-2010	DENV-1	I	MN628195
2010-2338	Lao PDR	Vientiane capital	Oct-2010	DENV-1	I	MN628196
2010-2342	Lao PDR	Vientiane capital	Oct-2010	DENV-1	I	MN628197
2010-2497	Lao PDR	Vientiane capital	Oct-2010	DENV-1	I	MN628198
2010-3196	Lao PDR	Vientiane capital	Dec-2010	DENV-1	I	MN628189
2010-3197	Lao PDR	Vientiane capital	Dec-2010	DENV-1	I	MN628191
2011-0010	Lao PDR	Vientiane capital	XX ^1^-2011	DENV-1	I	MN628185
2012-0005	Lao PDR	Vientiane capital	Apr-2012	DENV-1	I	MN628184
2012-0107	Lao PDR	Vientiane capital	Jul-2012	DENV-1	I	MN628200
2012-0113	Lao PDR	Vientiane capital	Jul-2012	DENV-1	I	MN628201
2012-0177	Lao PDR	Vientiane capital	Aug-2012	DENV-1	I	MN628199
2012-0210	Lao PDR	Vientiane capital	Aug-2012	DENV-1	I	MN628206
2012-0227	Lao PDR	Vientiane capital	Aug-2012	DENV-1	I	MN628204
2012-0258	Lao PDR	Vientiane capital	Sept-2012	DENV-1	I	MN628186
2012-0259	Lao PDR	Vientiane capital	Sept-2012	DENV-1	I	MN628205
2012-0260	Lao PDR	Vientiane capital	Sept-2012	DENV-1	I	MN628202
2012-0269	Lao PDR	Vientiane capital	Sept-2012	DENV-1	I	MN628203
2012-0331	Lao PDR	Vientiane capital	Oct-2012	DENV-1	I	MN628187
2012-0436	Lao PDR	Vientiane capital	Nov-2012	DENV-1	I	MN628188
2014-3216	Lao PDR	Vientiane capital	Sept-2014	DENV-1	I	MN628190
2015-3021	Lao PDR	Vientiane capital	May-2015	DENV-1	I	MN628211
2015-3048	Lao PDR	Vientiane capital	Jun-2015	DENV-1	I	MN628212
2015-3059	Lao PDR	Attapeu	Jul-2015	DENV-1	I	MN628213
2015-3062	Lao PDR	Attapeu	Jul-2015	DENV-1	I	MN628214
2015-3074	Lao PDR	Attapeu	Jan-2015	DENV-1	I	MN628215
2015-3080	Lao PDR	Attapeu	Apr-2015	DENV-1	I	MN628216
2015-3098	Lao PDR	Attapeu	Jul-2015	DENV-1	I	MN628217
2015-3122	Lao PDR	Attapeu	Aug-2015	DENV-1	I	MN628209
2015-3127	Lao PDR	Vientiane capital	Aug-2015	DENV-1	I	MN628218
2015-3135	Lao PDR	Attapeu	Aug-2015	DENV-1	I	MN628230
2015-3145	Lao PDR	Vientiane capital	Aug-2015	DENV-1	I	MN628219
2015-3173	Lao PDR	Attapeu	Sept-2015	DENV-1	I	MN628208
2015-3177	Lao PDR	Attapeu	Sept-2015	DENV-1	I	MN628207
2015-3178	Lao PDR	Attapeu	Sept-2015	DENV-1	I	MN628220
2015-3187	Lao PDR	Vientiane capital	Sept-2015	DENV-1	I	MN628221
2015-3189	Lao PDR	Attapeu	Sept-2015	DENV-1	I	MN628222
2015-3195	Lao PDR	Vientiane capital	Sept-2015	DENV-1	I	MN628223
2015-3204	Lao PDR	Vientiane capital	Sept-2015	DENV-1	I	MN628224
2015-3205	Lao PDR	Vientiane capital	Sept-2015	DENV-1	I	MN628210
2015-3213	Lao PDR	Vientiane capital	Sept-2015	DENV-1	I	MN628225
2015-3214	Lao PDR	Vientiane capital	Sept-2015	DENV-1	I	MN628226
2015-3228	Lao PDR	Vientiane capital	Sept-2015	DENV-1	I	MN628227
2015-3414	Lao PDR	Vientiane capital	Nov-2015	DENV-1	I	MN628228
2015-3416	Lao PDR	Vientiane capital	Nov-2015	DENV-1	I	MN628229
2016-3908	Lao PDR	Luangprabang	XX ^1^-2016	DENV-1	I	MN628231
2010-1947	Lao PDR	Vientiane capital	Sept-2010	DENV-2	Asian I	MN628249
2010-2108	Lao PDR	Vientiane capital	Sept-2010	DENV-2	Asian I	MN628250
2010-2340	Lao PDR	Vientiane capital	Oct-2010	DENV-2	Asian I	MN628251
2010-2498	Lao PDR	Vientiane capital	Oct-2010	DENV-2	Asian I	MN628245
2010-2500	Lao PDR	Vientiane capital	Oct-2010	DENV-2	Asian I	MN628246
2010-2723	Lao PDR	Vientiane capital	Oct-2010	DENV-2	Asian I	MN628242
2010-2724	Lao PDR	Vientiane capital	Oct-2010	DENV-2	Asian I	MN628233
2010-3023	Lao PDR	Vientiane capital	Nov-2010	DENV-2	Asian I	MN628243
2010-3024	Lao PDR	Vientiane capital	Nov-2010	DENV-2	Asian I	MN628244
2012-0136	Lao PDR	Vientiane capital	Aug-2012	DENV-2	Asian I	MN628252
2012-0181	Lao PDR	Vientiane capital	Aug-2012	DENV-2	Asian I	MN628237
2012-0237	Lao PDR	Vientiane capital	Sept-2012	DENV-2	Asian I	MN628232
2012-0254	Lao PDR	Vientiane capital	Sept-2012	DENV-2	Asian I	MN628234
2012-0265	Lao PDR	Vientiane capital	Sept-2012	DENV-2	Asian I	MN628247
2012-0309	Lao PDR	Vientiane capital	Oct-2012	DENV-2	Asian I	MN628248
2012-0388	Lao PDR	Vientiane capital	Nov-2012	DENV-2	Asian I	MN628236
2012-0404	Lao PDR	Vientiane capital	Nov-2012	DENV-2	Asian I	MN628240
2012-0423	Lao PDR	Vientiane capital	Nov-2012	DENV-2	Asian I	MN628235
2012-0425	Lao PDR	Vientiane capital	Nov-2012	DENV-2	Asian I	MN628238
2012-0449	Lao PDR	Vientiane capital	Nov-2012	DENV-2	Asian I	MN628239
2012-0468	Lao PDR	Vientiane capital	Dec-2012	DENV-2	Asian I	MN628241
2012-0226	Lao PDR	Vientiane capital	Aug-2012	DENV-3	II	MN628254
2016-3913	Lao PDR	Saravane	Jun-2016	DENV-3	III	MN628253
2013-2769	Lao PDR	Vientiane capital	Oct-2013	DENV-4	I	MN628258
2015-3131	Lao PDR	Vientiane capital	Aug-2015	DENV-4	I	MN628257
2016-3897	Lao PDR	Saravane	Jun-2016	DENV-4	I	MN628256
2016-3981	Lao PDR	Bolikhamxay	Jul-2016	DENV-4	I	MN628255

^1^ Sample number, defined by the year of collection and the order number of receipt at the laboratory. ^2^ Collection month unknown

## Data Availability

Nucleotide sequences of the complete E gene obtained in this study were submitted to EMBL-EBI and were available under accession no. from MN628181 to MN628258.
